# Dependency of crossover point on absorption changes in bilayer diffusion reflection measurements

**DOI:** 10.1117/1.JBO.29.8.087001

**Published:** 2024-08-28

**Authors:** Channa Shapira, Yuval Yedvav, Hamootal Duadi, Haim Taitelbaum, Dror Fixler

**Affiliations:** a Bar Ilan University, Faculty of Engineering, Ramat Gan, Israel; b Bar Ilan University, The Institute of Nanotechnology and Advanced Materials, Ramat Gan, Israel; c Bar-Ilan University, Department of Physics, Ramat Gan, Israel

**Keywords:** diffusion reflection, crossover point, absorption coefficient

## Abstract

**Significance:**

A better understanding of diffusion reflection (DR) behavior may allow it to be used for more noninvasive applications, including the development of *in vivo* non-damaging techniques, especially for medical topical diagnosis and treatments.

**Aim:**

For a bilayer opaque substance where the attenuation of the upper layer is larger than the attenuation of the lower layer, the DR crossover point ($C_{p}$) is location where the photons coming from the bottom layer start affecting the DR. We aim to study the dependency of the $C_{p}$ on absorption changes in different layers for constant scattering and top layer thickness.

**Approach:**

Monolayer and bilayer optical tissue-like phantoms were prepared and measured using a DR system. The results were compared with Monte Carlo simulations.

**Results:**

There is an agreement between the experiments and the simulations. $C_{p}$ correlates with the square root of the absorption coefficient ratio of the lower layer to the top layer.

**Conclusion:**

The experimental findings support and validate the theoretical prediction describing the dependency of the $C_{p}$ on the square root of the ratio of the layers’ absorption coefficients. In addition, a secondary breaking point is suggested to be observed experimentally at the entrance to the noise area.

## 1 Introduction

Diffusion reflection (DR)-based techniques are methods for extracting the optical properties of a given turbid sample based on measuring the diffused reflectance. These optical properties, such as scattering (represented by the reduced scattering coefficient $\mu_{s}^{\prime }$) and absorption (represented by the absorption coefficient $\mu_{a}$), are wavelength-dependent.^1^ For different materials, the optical properties are unique and yield distinctive appearance features accordingly. Therefore, acquiring the optical properties of an unidentified sample enables the identification of its material. In the context of biological samples, the optical properties may contain valuable information regarding the medical condition of the specimen.^2^^,^^3^ DR-based techniques are adept at extracting optical properties while maintaining simplicity, cost-effectiveness, and minimal energy usage, and they are noninvasive and safe for biological tissues. Consequently, such techniques have been used for various applications, such as in the medical field,^4^^–^^7^ food analysis,^8^ and more.^9^ Using DR-based techniques, one may utilize the crossover point ($C_{p}$) occurring in layered opaque samples, when the deeper layers start affecting the light reemitted from the sample. The $C_{p}$ depends on the optical properties of the layers, as well as their thickness. Knowing the relation between the physical and optical properties and the location of the $C_{p}$ allows one to extract more parameters, such as the thickness of the top layer, from the same measurement. Thus, the $C_{p}$ has a clinical significance. The $C_{p}$ was used for the depth detection of elements within soft tissues: it was used for detecting tattoos’ type and depth,^10^ and it holds the potential to be useful for profiling permeation of nanoparticles among skin layers. Theoretical research predicted the relationship between the $C_{p}$ and the sample properties, and the current research aims to study this relationship experimentally and support the theoretical prediction.

**Fig. 1:**
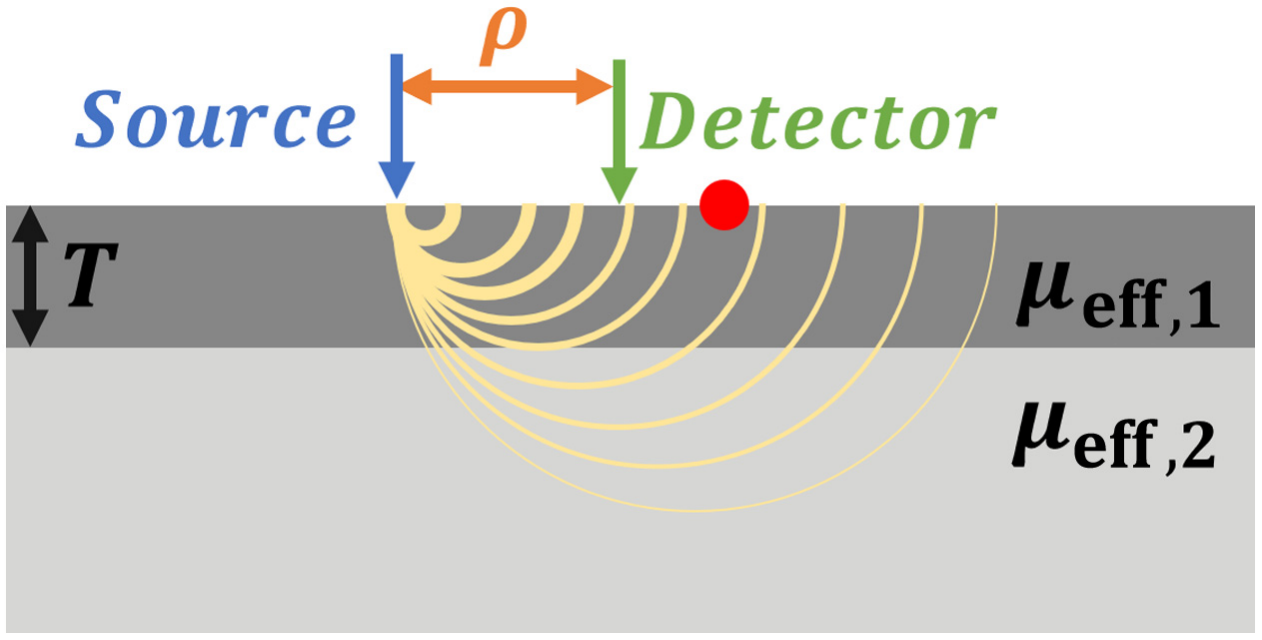
Bilayer sample model in a DR system. The bilayer model includes a thickness T of the first (upper) layer, and each layer has its own optical property, $\mu_{\mathrm{eff},1}$ and $\mu_{\mathrm{eff},2}$ for the top and bottom layers, respectively. The sample is illuminated using the source fiber (denoted by a blue arrow), and a detector (denoted by a green arrow) moving along a radial axis ρ collecting the diffused reflectance at each point. Some photon paths are illustrated by yellow curves. For small ρ values, photons depart the sample after passing through the top layer only. At very large ρ values, most photons passed through both layers. The $C_{p}$, denoted by a red dot, is the initial location where photons departing the sample pass through both layers.

Traditionally, DR considers the sample as a semi-infinite homogeneous substance with an effective attenuation coefficient^11^

$$\mu_{\mathrm{eff}}=\sqrt{3\mu_{a}(\mu_{s}^{\prime }+\mu_{a})}$$ (1)

that can be extracted. Therefore, given one of the two properties, $\mu_{a}$ or $\mu_{s}^{\prime }$, the other can be deduced. The measurement is simple: the sample is illuminated, and the detector captures the light emitted from the sample along the radial axis extending outward from the center of illumination. The collected light contains the diffuse reflectance R, i.e., the light that penetrated the tissue and scattered before leaving the sample through its top surface, which is the air-sample interface. In Fig. 1, the top surface is the top border of the top layer.

Analyzing the results, R is described as a function of the distance along the radial axis, ρ. In the multiple scattering area, where ρ is larger than $1/\mu_{s}^{\prime }$, the diffusion approximation is eligible for describing light propagation in tissue. The solution for the diffusion equation allows R(ρ) to be approximated and simplified to an exponential decrease^7^^,^^12^^,^^13^

$$R(\rho )=(\frac{C_{1}}{\rho^{m}})\exp(-\mu_{\mathrm{eff}}\cdot \rho ),$$ (2)

where $C_{1}$ is an empirically determined parameter depending on the optical properties and m is a constant depending on the detection range and also on the optical properties.^7^^,^^12^ It was shown that for $\mu_{a}\approx 0$, the appropriate value is m=2, and that for very high absorption is m≈0;^14^ however, it was also shown that the value m=1 is appropriate for a detection range of 2 to 10 mm for varying absorption values. There are researchers, however, who used the values m=0.5^12^^,^^15^ or m=2.^13^^,^^16^

Regarding a measurement in the form of Eq. (2), it is useful to use

$$\ln(R(\rho )\cdot \rho^{m})=-\mu_{\mathrm{eff}}\cdot \rho +C$$ (3)

rather than R(ρ) itself. For a bulk substance, the equation yields a graph with a region that is approximately linear with a slope that equals the effective attenuation coefficient ($\mu_{\mathrm{eff}}$) of the substance.^7^

The described DR technique is used for various applications. However, addressing every sample as a bulk substance is somewhat simplistic. Therefore, the diffused reflectance was also studied for bilayer substances.^17^^,^^18^ Figure 1 shows such a model: The bilayer model includes a thickness T of the first (upper) layer, and each layer has its own optical property, $\mu_{\mathrm{eff},1}$ and $\mu_{\mathrm{eff},2}$ for the top and bottom layers, respectively. The sample is illuminated using the source fiber (denoted by a blue arrow) and a detector (denoted by a green arrow) moving along a radial axis ρ collecting the diffused reflectance R(ρ) at each point. Possible photon paths are illustrated by yellow curves.

A simpler version of this model, in which both layers have the same scattering but differ in their absorption, was studied theoretically by Taitelbaum et al.^18^ based on the random walk model and Dayan et al.^19^ using a diffusion approximation model. It was shown that if the top layer has greater absorption than the bottom layer, the output graph of R(ρ) can be sectioned into two regions: In the first region, at small ρ values, photons depart the sample after passing through the top layer only. In the second region, at larger ρ values, the vast majority of the photons passed through both layers. The $C_{p}$, denoted by a red dot, is the initial location where photons departing the sample pass through both layers. In the illustration, the yellow curves arriving at the surface close to the source, before the $C_{p}$, pass through the top layer only, whereas the yellow curves arriving, at distances larger than $C_{p}$, pass through both layers.

In the DR graph described as ln(R(ρ)·ρ), the $C_{p}$ separates the two regions, which have distinct behaviors: the slopes of the first and second regions fit $\mu_{\mathrm{eff},1}$ and $\mu_{\mathrm{eff},2}$, respectively. Taitelbaum et al.^18^ predicted theoretically that under the circumstances of similar scattering for both layers, the point $C_{p}$ that separates the regions behaves as

$$C_{p}\propto T(1+\sqrt{\frac{\mu_{a,2}}{\mu_{a,1}}}).$$ (4)

The physical meaning of this result is that the location of the crossover point is linearly dependent on the upper layer thickness, with a pre-factor consisting of two terms. The first is the short-distance contribution (dominated by the upper layer attenuation), and the second term is the long-distance contribution where the bottom layer (with $\mu_{a,2}$) becomes dominant over the $\mu_{a,1}$ of the upper layer, hence the term $\sqrt{\mu_{a,2}/\mu_{a,1}}$. Dayan et al.^19^ presented an equivalent relation resulting from the diffusion approximation, which also predicted that $C_{p}$ increases when the ratio $\sqrt{\mu_{a,2}/\mu_{a,1}}$ increases. Following these theoretical models, the existence of the $C_{p}$ was obtained experimentally by Ankri et al.,^20^ who also showed qualitatively that the $C_{p}$ shifts together with the ratio $\sqrt{\mu_{a,2}/\mu_{a,1}}$. Later, Rudraiah et al.^10^ showed experimentally that $\mu_{\mathrm{eff}}$ of the different layers can be extracted from the slopes. However, Rudraiah et al.^10^^,^^21^^,^^22^ claimed to find empirically a different behavior of the $C_{p}$. Therefore, the purpose of the current research is to study systematically the dependency of $C_{p}$ on the absorption coefficients of the two layers.

This research includes Monte Carlo (MC) simulations of the diffused reflectance of bilayer phantoms with fixed $\mu_{s}^{\prime }$ and top layer thickness T but varying $\mu_{a}$ for the top and bottom layers. In addition, the research includes optical phantoms made with the same optical properties as the simulation to verify the location of $C_{p}$ found in the MC simulations. The research aims to study empirically the behavior of $C_{p}$ and discuss the results of Rudraiah et al.^10^^,^^21^^,^^22^

## 2 Materials and Methods

### 2.1 Monte Carlo Simulation

A Monte Carlo (MC) simulation is a computational method allowing for the analysis of complex processes or phenomena through repeated random trials. The simulation uses random sampling to model the behavior of a system, which is, in this case, the trend of the diffused reflection of optical phantoms illuminated by a 700 nm source. All the phantoms have T=2  mm and anisotropy factor g=0.69 and $\mu_{s}=2.9\;{\mathrm{mm}}^{-1}$, resulting in $\mu_{s}^{\prime }=0.89\;{\mathrm{mm}}^{-1}$. The optical properties input for the simulation were carefully chosen to fit the experiment, including optical phantoms, as will be discussed below.

In this work, the MC simulation ran over $10^{9}$ photons and tested semi-infinite samples. There were two types of simulations. The first type involved simulating a uniform semi-infinite medium. Six simulations as such were applied with varying absorption coefficients and the same scattering coefficients. The second type of simulation examined a semi-infinite medium with a T=2  mm layer on top. The layers differ by their absorption coefficient, whereas their scattering coefficient is similar. The bilayer simulations were designed based on the six absorption coefficient values from the uniform-media simulation: the top layers had three higher absorption values, and the bottom layers had three lower absorption values; therefore, there were nine bilayer simulations. The optical properties of the simulation were chosen to fit the optical phantoms used later in the experiment. The scattering properties of the phantoms are detailed in Fig. S1 in the Supplementary Material, and the $\mu_{a}$ values of the different phantoms can be found in Table S2 in the Supplementary Material.

### 2.2 Optical Setup

**Fig. 2:**
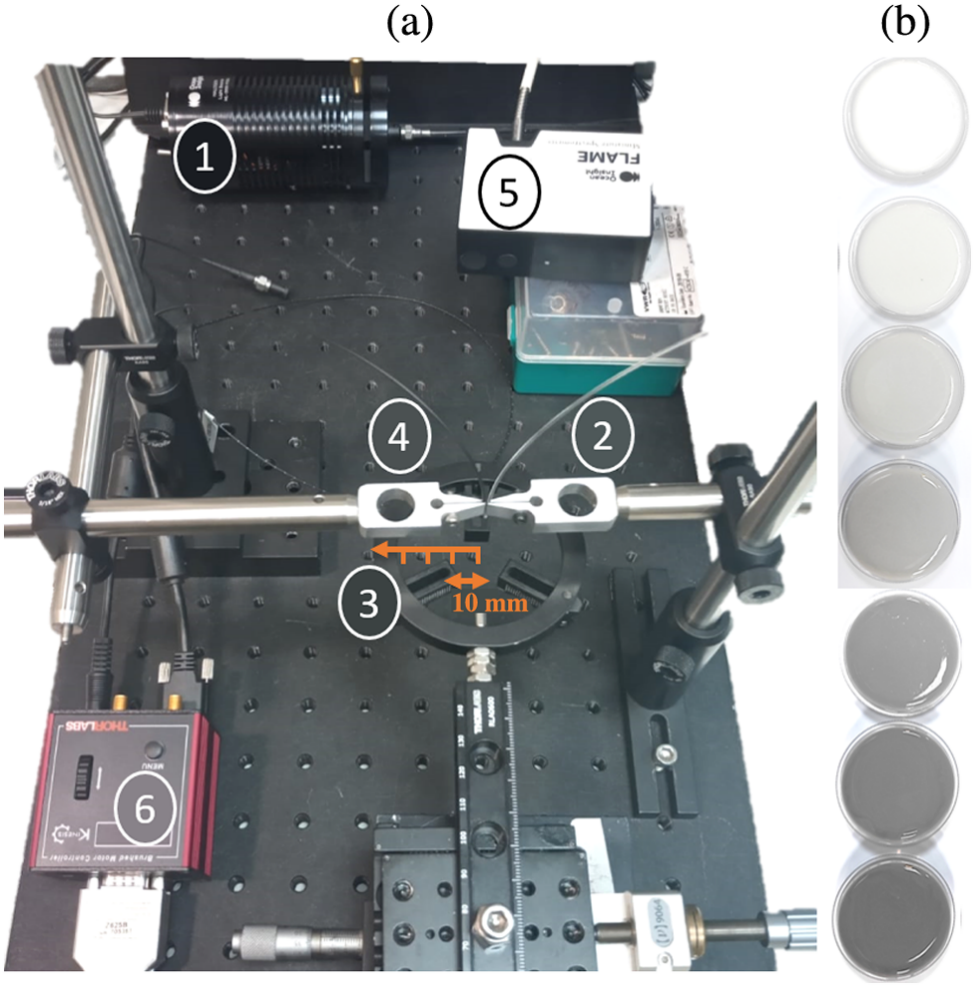
(a) DR optical setup. Light travels from the light source (1) through the source fiber (2) and illuminates a sample that is fixed on the sample holder (3). The light is collected by the detector fiber (4) to a spectrometer (5) in distances of 0.2 mm on a radial axis, denoted with an orange arrow. The motion of the detector fiber is controlled using a motor (6). (b) Seven single-layer optical phantoms with constant scattering and varying absorption. The single-layer phantoms have a 53 mm diameter, 10 mm height, and scattering coefficient $\mu_{s}^{\prime }=0.89\;{\mathrm{mm}}^{-1}$. The absorption coefficients are $\mu_{a}=0$, 0.03, 0.09, 0.12, 0.24, 0.41, and $0.6\;{\mathrm{mm}}^{-1}$. The six absorbing phantoms were used in the experiment, and the seventh is an additional control phantom without ink addition.

The experimental setup of the DR technique is presented in Fig. 2. The light source [Fig. 2(a.1)] is a tungsten-halogen lamp (HL-2000-HP-FHSA, Ocean Insight, with 20 W output power). The light enters the source fiber 0.63 numerical aperture, core diameter of 1.5 mm, Prismatic Ltd., Alton, United Kingdom, [Fig. 2(a.2)] that illuminates a sample that is fixed on a sample holder [Fig. 2(a.3)]. The light is collected by the detector fiber [0.63 numerical aperture, core diameter of 1 mm, Prismatic Ltd.; Fig. 2(a.4)] and measured using a spectrometer [FLAME-T-VIS-NIR-Spectrometer, Ocean Insight; Fig. 2(a.5)]. The measurement is taken along a radial axis (denoted with an orange arrow) from the center of illumination, within a radial distance of 2 to 12 mm every 0.2 mm. The motion of the detector fiber is controlled using a motor [Fig. 2(a.6)]. The samples in the study are optical phantoms with a 53 mm diameter and 10 mm height [Fig. 2(b) shows monolayer phantoms with constant scattering and varying absorption].

**Fig. 3:**
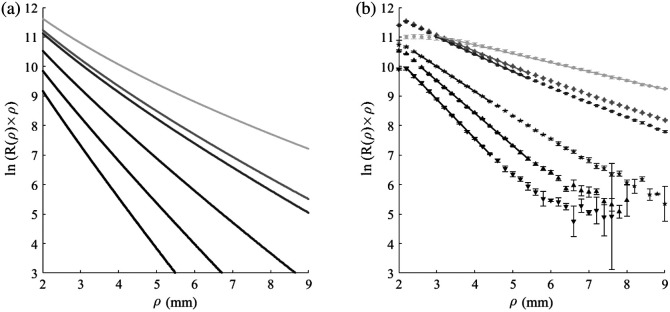
Single layer phantoms measurements. (a) Results of MC simulation, simulating monolayer phantoms with the optical properties of the actual phantoms. (b) The measurements of the phantoms are presented according to the absorption coefficients: $\mu_{a}=0.03$, 0.09, 0.12, 0.24, 0.41, and $0.6\;{\mathrm{mm}}^{-1}$. The darker the color, the higher the absorption. As the absorption increases, the slope is steeper. The simulation shows a similar behavior to the experiment.

**Fig. 4:**
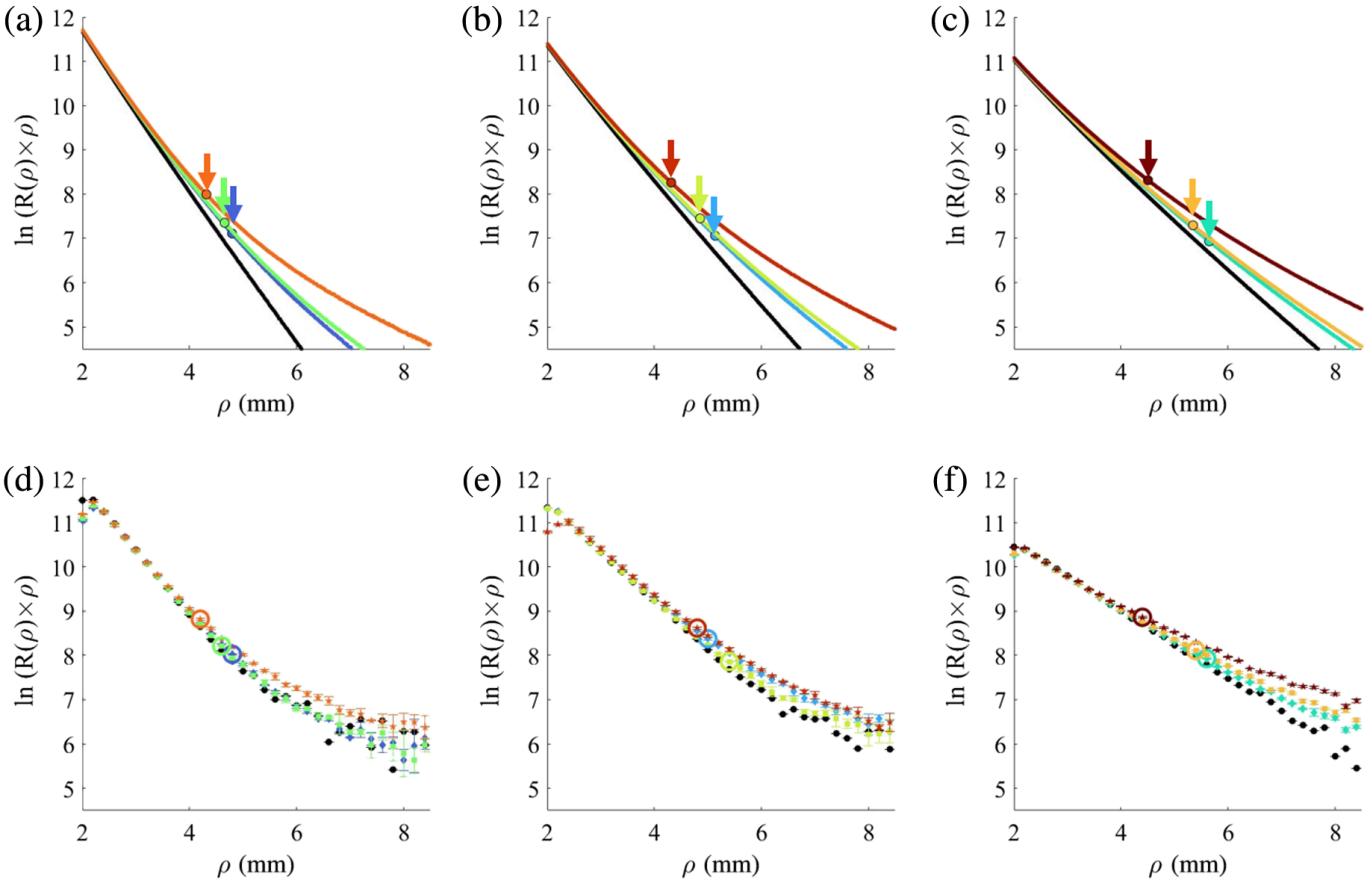
Diffused reflectance results for bilayer phantoms, simulation and experiments. For both simulation and experiment graphs, the corresponding phantom is represented by the same color. Each figure compares three bilayer phantoms with an identical top layer $\mu_{a,1}$ and with different bottom layers $\mu_{a,2}=0.03$, 0.09, and $0.12{\;\mathrm{mm}}^{-1}$ in addition to the monolayer phantom (black) with the optical properties of the mutual top layer. Panels (a), (b) and (c) show simulations of phantoms with $\mu_{a,1}=0.6$, 0.41, and $0.24{\;\mathrm{mm}}^{-1}$, respectively. Panels (d), (e), and (f) show experimental results of phantoms with $\mu_{a,1}=0.6$,  0.41, and $0.24{\;\mathrm{mm}}^{-1}$, respectively.

**Fig. 5:**
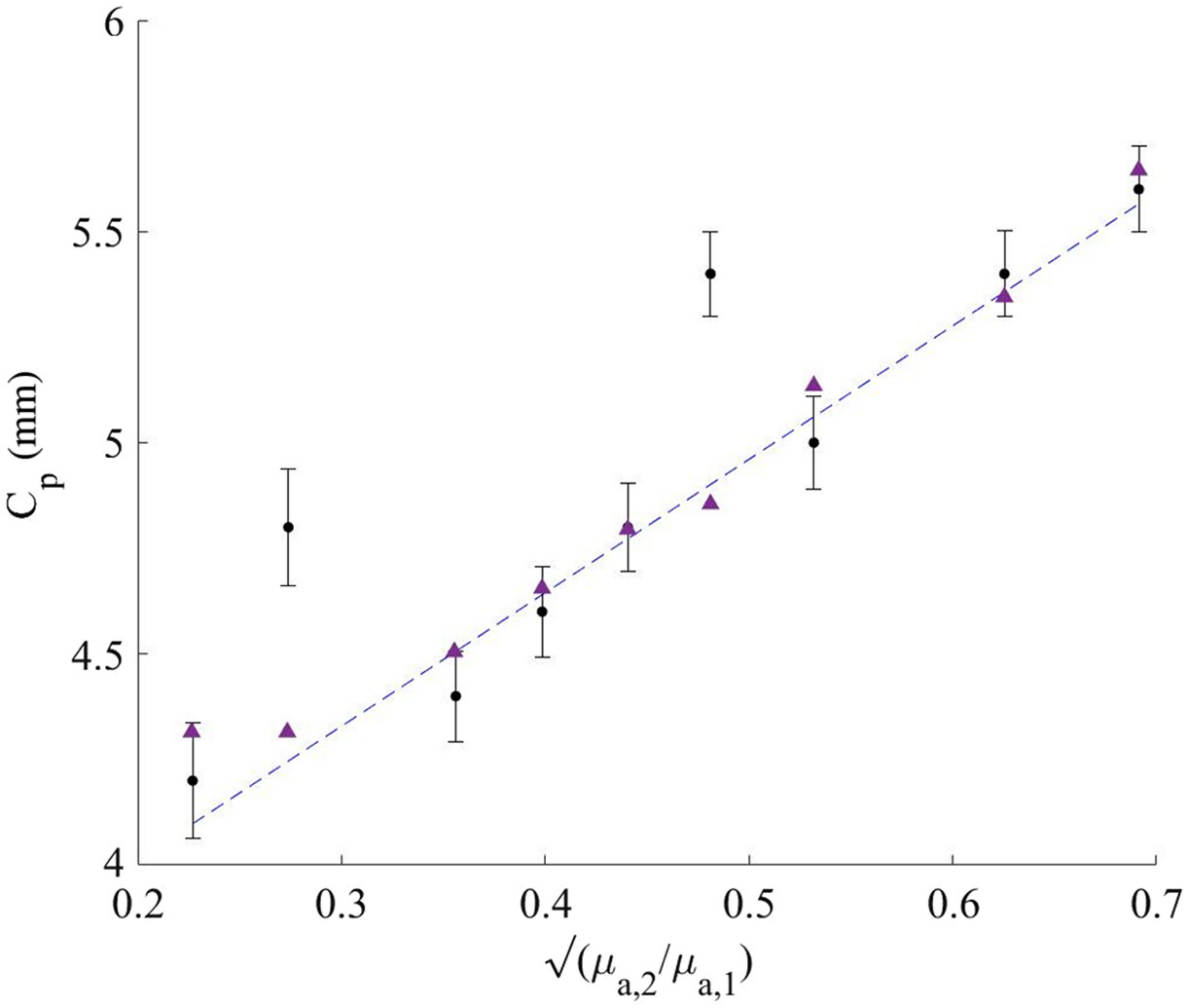
$C_{p}$ as a function of $\sqrt{\mu_{a,2}/\mu_{a,1}}$. The $C_{p}$ extracted from experiments and simulation results are represented by black dots and purple triangles, respectively. The dashed line is the linear fit of the experimental data, excluding the exceptional points.

**Fig. 6:**
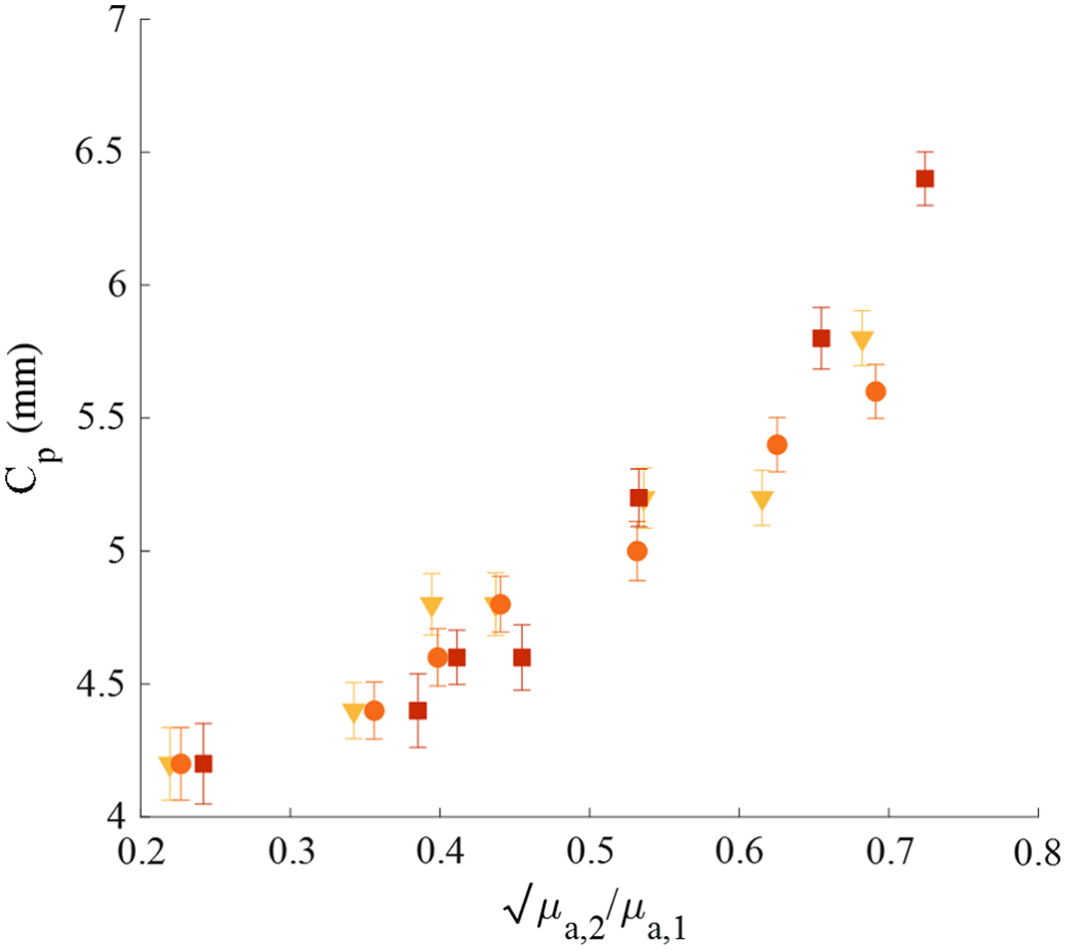
$C_{p}$ as a function of $\sqrt{\mu_{a,2}/\mu_{a,1}}$. at three wavelengths: λ=650, 700, and 750 nm represented by yellow triangles, orange circles, and red squares, respectively, excluding the exceptional points.

### 2.3 Optical Phantoms

The experiments in this research were performed using optical phantoms. The ability to prepare the phantoms with controlled optical properties makes them suitable for the aim of the experiment: validation of the theoretical model regarding the effect of the absorption of the different layers on the resulting $C_{p}$ value. The phantoms contain Intralipid (IL) (Intralipid 20% Emulsion, Sigma-Aldrich, Jerusalem, Israel), double desterilized water (DDW), ink, and agar (Agarose-low gelling temperature, Sigma-Aldrich). The percentages of IL and ink set the scattering and absorption properties of each phantom, respectively. The agar is melted within the DDW on top of a hotplate and then mixed with the other ingredients. Last, the final solution is poured into Petri dishes with 53 mm diameter and 10 mm height to solidify overnight. Bilayer samples are made in two phases: first, the 8 mm thick bottom layer is prepared, and after it solidifies, the top layer can be made as well, pouring the solution of the top layer (2 mm) on top of the solid bottom layer and again, leaving the samples to solidify overnight.

The optical properties of a phantom are derived from its ingredients: the IL causes scattering, and the ink causes absorption. The relation between the IL and the scattering coefficient was shown by Van Staveren et al.^23^ The agar ingredient in the phantoms aims for their solidification; however, it affects their scattering as well. Following Cubeddu et al.,^24^ for solid phantoms with the addition of agar, the scattering coefficient should be multiplied by 0.7.

In the described experiment, $\mu_{s}^{\prime }$ was fixed for all the phantoms, as well as the top layer thickness of 2 mm, whereas $\mu_{a}$ varies. Thus, all the phantoms contained 1.1% IL, and so, $\mu_{s}^{\prime }(\lambda )$ was calculated accordingly. At 700 nm, the scattering properties of all the phantoms are designed to be: g=0.69, $\mu_{s}=2.9\;{\mathrm{mm}}^{-1}$, $\mu_{s}^{\prime }=0.89\;{\mathrm{mm}}^{-1}$. Full data regarding wavelengths 650 and 750 as well can be found in Table S1 in the Supplementary Material.

The absorption was set according to the amount of ink in each phantom. The ink concentrations of the six monolayer phantoms and the designed $\mu_{a}$ at 700 nm can be found in Table S2 in the Supplementary Material. Three phantoms were designated as low-absorption phantoms, and three phantoms were designated as high-absorption phantoms. In addition, nine bilayer phantoms were prepared based on the $\mu_{a}$ values of the monolayer phantoms. The nine phantoms are composed of all the variations of the high-absorption values at the top layer and low-absorption values at the bottom layer, as detailed in Table S3 in the Supplementary Material.

### 2.4 C_p_ Extraction

To extract the $C_{p}$ of each bilayer phantom, the bilayer phantom should be compared with the relevant monolayer phantom. A bilayer phantom having $\mu_{a,1}$ at the top layer and $\mu_{a,2}$ at the bottom layer should be compared with a monolayer phantom with $\mu_{a,1}$. As both simulation and measurement are presented following Eq. (3) for m=1, the $C_{p}$ would be the point where the graphs separate. However, to study the influence of the optical properties of the top layer on the location of the $C_{p}$, a method for extracting the $C_{p}$ should be formulated. In this research, the chosen method is implemented as an algorithm. The algorithm compares the local slope of the bilayer phantom and the slope of the relevant monolayer phantom (with $\mu_{a,1}$). The $C_{p}$ is chosen to be the point where the absolute difference between the slopes is larger than α of the slope of the monolayer phantom, following Eq. (5) where $M_{\text{bilayer}}$ is the local slope of the bilayer phantom graph and $M_{\text{monolayer}}$ is the slope of the monolayer phantom graph.

$$|M_{\text{bilayer}}-M_{\text{monolayer}}|>\alpha \cdot |M_{\text{monolayer}}|.$$ (5)

The constant α is chosen according to the sensitivity of the experimental setup. For the simulation, α would be the same. However, due to the smooth nature of the simulation, as opposed to the noisy nature of the experiment, the point is not necessarily found in the same location but may have an additional DC level. The results are shown for wavelength of λ=700  nm. A comparison of experimental results of λ=650, 700, and 750 nm is presented in Figure S1 in the Supplementary Material.

## 3 Results

The first part of the results refers to monolayer phantoms with fixed scattering and varying absorption. MC simulations were applied to model the diffused reflectance of the phantoms in the form described in Eq. (3) for m=1. The simulation’s results are presented in Fig. 3(a). The grayscale colors of the graphs correspond to the absorption of the measured phantom, meaning that the highest absorption is represented by the darker color and the smallest absorption by the lightest color. The results show that higher absorption yields steeper slopes. As expected, a resembling behavior was observed in experiments measuring the IL optical phantom [Fig. 3(b)]. The results are shown for the wavelength of λ=700  nm. Additional full-length experimental results at λ=650, 700, and 750 nm are presented in Fig. S1 in the Supplementary Material. Note that there are differences between the simulation and experimental results. The simulation does not profess to exactly present the experimental reality. The simulation assumes given optical parameters such as, $\mu_{a}$, $\mu_{s}$, and g and is not capable for considering the different particles in every interaction. The simulation also does not take into account the numerical aperture, the distance from the source to the air-sample interface, and the detector size, factors that are known to affect DR measurements.^25^ Nevertheless, the trend of the simulations resembles the trend of the experiments.

The second part of the results refers to bilayer optical phantoms. These nine phantoms consist of an 8 mm thick bottom layer with low absorption values and a 2 mm thick top layer with high absorption values. The absorption values are similar to those of the six monolayer phantoms. Table S2 in the Supplementary Material shows the ink concentration in the different phantoms, and Table S3 in the Supplementary Material details the bilayer phantom composition of the monolayer phantoms. The results are presented in Figs. 4(a)–4(c). Each plot presents simulations of three phantoms with the same top-layer absorption and varying bottom-layer absorption. In each plot, the black line represents the monolayer simulation corresponding to the absorption of the top layer, which is identical to the three bilayer phantoms depicted in the plot. For all the plots, where ρ is small, the graphs have similar slopes due to the common top 2 mm. As ρ increases, the influence of the bottom layer becomes apparent, and the slopes change according to the absorption of the bottom layer. For each graph, the $C_{p}$ is marked with a dot and an arrow colored by the same color of the graph. The results of the experiment are presented in Figs. 4(d)–4(f), where each phantom is represented by the same color as in the simulation plot. For each graph, the $C_{p}$ is marked with a circle with the same color of the graph. A visual comparison between simulation and experiments shows similar $C_{p}$ positions. The presented results are shown for the wavelength of λ=700  nm. Additional full-length experimental results at λ=650, 700, and 750 nm are presented in Figs. S2–S4 in the Supplementary Material, respectively.

The values of the $C_{p}$ are shown in Fig. 5, where the experimental and simulation results are represented by black dots and purple triangles, respectively. The dashed line is the linear fit of the experimental data, excluding the exceptional points. The equation of the linear fit is

$$C_{p}=3.2\cdot \sqrt{\frac{\mu_{a,2}}{\mu_{a,1}}}+3.4=3.2\cdot (\sqrt{\frac{\mu_{a,2}}{\mu_{a,1}}}+1)+0.2.$$ (6)

The $R^{2}$ of the experimental results excluding the two exceptional points and the linear fit is $R^{2}\approx 0.98$. The $R^{2}$ of the linear fit and the simulation results is $R^{2}\approx 0.99$. Hence, the simulation and experimental results agree and clearly support the theoretical result presented by Taitelbaum et al.,^18^ as shown in Eq. (4). The additive 0.2 mm constant could be attributed to the chosen method for extracting the $C_{p}$, yet it well obeys the linear proportionality of Eq. (4). The experimental results showing the $C_{p}$ values extracted at 650, 700, and 750 nm are presented in Fig. 6. The experimental results converge to the same trendline with $R^{2}\approx 0.91$. This could be explained by the fact that the ratio between the absorption coefficients is independent of the wavelength. A comparison separating the different wavelengths is presented in Fig. S5 in the Supplementary Material.

## 4 Discussion

As detailed in Sec. 1, the theoretical models presented by Taitelbaum et al.^18^ and Dayan et al.,^19^ predicted the behavior of $C_{p}$, as presented in Eq. (4). The preliminary findings of Ankri et al.^20^ and the results of the current research, presented in Eq. (6), support it as well, for a wide range of parameters. However, Rudraiah et al.^10^^,^^21^^,^^22^ found experimentally that the $C_{p}$ has a different behavior. A mindful reader may notice some differences between the theoretical models and the experiment handled by Rudraiah et al. First, the theoretical model describes bilayer media, whereas in the presented experiments, there is a thin layer of ink in between, making the sample a tri-layer medium. The middle layer is either ink^10^ in the phantoms research or a tattoo in the *ex vivo* research.^10^^,^^22^ Second, Rudraiah et al.^10^^,^^22^ presented an equation describing the behavior of $C_{p}$ as a function of the absorption coefficient of the different layers. Their equation does not depend on the scattering property, although, in fact, the bilayer samples in their work differ also in their scattering properties,^10^^,^^22^ which can significantly change the location of the $C_{p}$. In another phantom-experiment work, Rudraiah et al.^21^ measured phantoms with constant scattering and varying absorption, as described in the theoretical models. Rudraiah et al. showed a point where the graph of the bilayer measurements breaks, which Rudraiah et al. notated as the $C_{p}$. It is important to notice that the point presented in their work is neither the point presented in this work nor in the theoretical models. The $C_{p}$ discussed in the theoretical model separates between the top layer region, closer to the source, and the bottom layer region at larger distances. Rudraiah et al. reported that the slope before the point yields the optical properties of the bottom layer and the slope after the point does not correlate with any of the layers’ optical properties. The meaning of the breaking point found by Rudraiah et al. was a region that can no longer be described by the simplified equation in Eq. (2) for m=1. As such, it describes an interesting intersection point in Rudraiah et al.’s experiments but not the well-defined crossover point $C_{p}$ in bi-layered media given in Eq. (4).

**Fig. 7:**
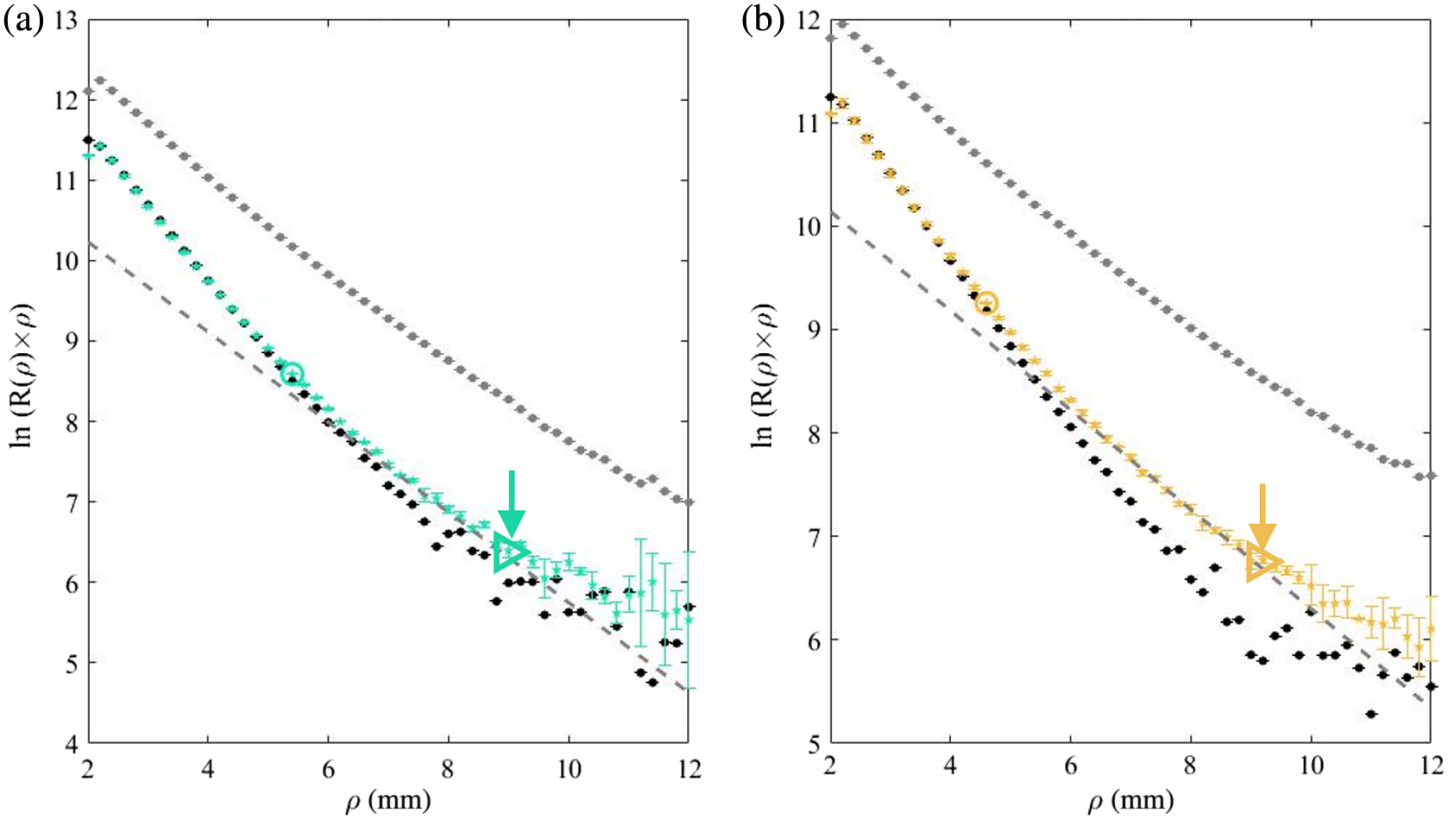
Two types of breaking points on bilayer phantoms’ graphs. In both graphs, the colored stars represent a bilayer measurement [of phantoms with $\mu_{a1}=0.24\;{\mathrm{mm}}^{-1}$ and $\mu_{a,2}=\;0.12$, $0.09{\;\mathrm{mm}}^{-1}$ in panels (a) and (b), respectively], whereas the black and gray circles represent the monolayer phantom of the top and bottom layers, respectively. The dashed gray lines have the slope of the bottom layer trend line. The hollow colored circles indicate the location of the $C_{p}$, separating between the top and bottom layer regions. The hollow right-pointing triangles designate the second type of breaking point of the bilayer graphs.

In the current research, a second breaking point after the second region is observed as well, as shown in Fig. 7. Figures 7(a) and 7(b) present plots, in which colored stars represent the bilayer measurement of phantoms with $\mu_{a,1}=0.24\;{\mathrm{mm}}^{-1}$ and $\mu_{a,2}=\;0.12$, $0.09{\;\mathrm{mm}}^{-1}$, respectively. The black and gray circles represent the monolayer phantom of the top and bottom layers, respectively. The dashed gray lines have the slope of the bottom layer trend line. The figure shows two types of breaking points on bilayer phantoms’ graphs. The first type, marked with a ring, is the $C_{p}$, the crossover point given by Eq. (4), separating between the top and bottom layer regions. The second breaking point is marked with a triangle at ρ=8.8  mm and ρ=9.2  mm at Figs. 7(a) and 7(b), respectively. These values correlate with five transport mean free path (MFP′) distances of the bottom layer optical properties. The value of five MFP′ is known for the noise limit^26^ and $\mathrm{MF}P^{\prime }=1/\mu_{\mathrm{eff}}$ where $\mu_{\mathrm{eff}}$ is presented in Eq. (2). The values of five MFP′ calculated for the presented phantoms are ρ≈8.4  mm and ρ≈9.4  mm at Figs. 7(a) and 7(b), respectively.

## 5 Summary and Conclusions

This research studies the phenomenon of the crossover point, occurring in bilayer structured media. The phenomenon was modeled theoretically using random walk and diffusion approximation methods for similar scattering and varying absorption in the two layers of a substance. After the theoretical model of the behavior was proposed, different researchers demonstrated the existence of the point and used it to extract the optical properties of turbid samples. The $C_{p}$ allows one to detect not only the effective attenuation coefficient of the sample’s layers but also the thickness of the top layer. Thus, it can be used for detecting the depth of absorbing elements within tissues, as was done previously for detecting tattoo type and depth. In addition, the technique has the potential to be used for profiling nanoparticle permeation among tissue layers. This work methodologically tested the behavior of the $C_{p}$ as a function of the ratio between the absorption coefficients of the layers with a fixed top layer thickness and supports, with both simulation and experiment, the theoretical results.

## 6 Associated Content

Supplementary Material: The supplementary material includes the following sections:

1. Optical properties of solid phantoms.
2. Full-length measurements at 650, 700, and 750 nm.
3. Extracted $C_{p}$ at 650, 700, and 750 nm.

## Supplementary Material



## Data Availability

The data presented in this article are publicly available in FigShare at https://doi.org/10.6084/m9.figshare.25532881.v1.
