# Supplementary material for: Dependency of crossover point on absorption changes in bilayer diffusion reflection measurements
Source: J Biomed Opt. 2024 Aug 28;29(8):087001. doi: 10.1117/1.JBO.29.8.087001 (PMC11350519; doi:10.1117/1.JBO.29.8.087001)
Supplement: Supplementary file 1 [file JBO_029_087001_SD001.docx]

**Supplementary materials: Dependency of crossover point on absorption changes in bilayer diffusion reflection measurements**

*Channa Shapira^1,2^, Yuval Yedvav^1^, Hamootal Duadi^1,2^, Haim Taitelbaum^3^, Dror Fixler^1,2,^**

*^1^Faculty of Engineering, Bar Ilan University, Ramat Gan 5290002, Israel*

*^2^The Institute of Nanotechnology and Advanced Materials, Bar Ilan University, Ramat Gan 5290002, Israel*

*^3^Department of Physics, Bar-Ilan University, 52900 Ramat-Gan, Israel*

1. **OPTICAL PROPERTIES OF SOLID PHANTOMS**

The optical properties of the used solid phantoms derives from their ingredients. The scattering derives from the percentages of intralipid, agar and water in the sample. In the phantoms prepared for the experiments the concentration of these ingredients was constant and therefore the scattering properties of the phantoms were also constant. The scattering properties of the phantoms at wavelength $\lambda=650, 700, 750 [nm]$ is detailed in Table 1.

**Table 1. Scattering properties of optical phantoms.**

| $\lambda$ [nm] | $g$ | $\mu_{s} [mm^{-1}]$ | $\mu_{s}^{'} [mm^{-1}]$ |
| --- | --- | --- | --- |
| 650 | 0.72 | 3.47 | 0.96 |
| 700 | 0.69 | 2.9 | 0.89 |
| 750 | 0.67 | 2.46 | 0.82 |

The absorption derives from the amount of ink at every phantom. The designed $\boldsymbol{\mu}_{\boldsymbol{a}}$at 700 nm and the ink concentrations in the monolayer phantoms are presented in Table 2. Phantoms 1L1, 1L2, 1L3 have lower absorption and are later used for the bottom layer at the bilayer phantoms. Phantoms 1L4, 1L5, 1L6 have higher absorption and are later used for the top layer at the bilayer phantoms.

Table 2. monolayer phantom $\boldsymbol{\mu}_{\boldsymbol{a}}\boldsymbol{[m}\boldsymbol{m}^{\boldsymbol{-1}}\boldsymbol{]}$ at 700 nm and ink concentration.

| sample name | $\mu_{a} \left[ mm^{-1} \right]$ at 700nm | ink concentration [x10^-4^] |
| --- | --- | --- |
| 1L1 | 0.12 | 1.1 |
| 1L2 | 0.09 | 0.6 |
| 1L3 | 0.03 | 0.2 |
| 1L4 | 0.6 | 5.4 |
| 1L5 | 0.41 | 3.2 |
| 1L6 | 0.24 | 2.1 |

1. **FULL LENGTH DR MEASUREMENTS AT 650nm, 700nm, AND 750nm**

The monolayer full-length measurements are presented in Figure 1 for 650nm (Figure 1 (a)), 700nm (Figure 1 (b)) and 750nm (Figure 1 (c)). As the absorption is higher, the ink concentration is higher, and therefore the absorption is higher as well. It can be seen that as the absorption is more significant, the signal is lost earlier.


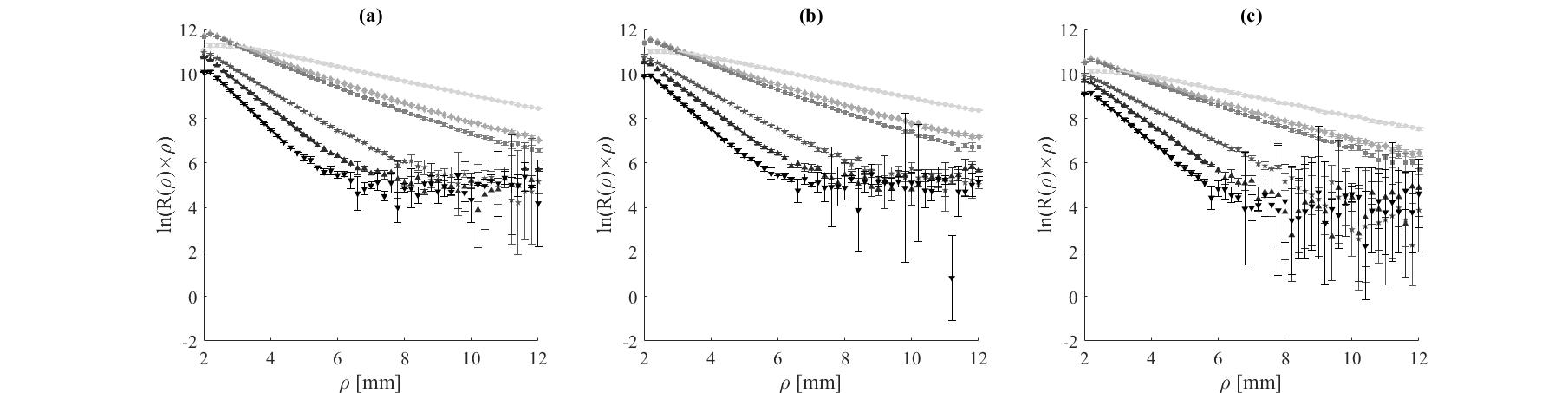


Figure 1. Monolayer measurements for (a) 650nm, (b) 700nm, and (c) 750nm.

The bilayer phantoms were made from the batches prepared for the monolayer phantoms as detailed in Table 3.

Table 3. Monolayer phantoms composition in bilayer phantoms.

| bottom\top | 1L4 | 1L5 | 1L6 |
| --- | --- | --- | --- |
| 1L1 | 2L1 | 2L2 | 2L3 |
| 1L2 | 2L4 | 2L5 | 2L6 |
| 1L3 | 2L7 | 2L8 | 2L9 |

The bilayer full-length results at 650nm, 700nm and 750nm are presented in Figure 2, Figure 3, and Figure 4, respectively. In every figure, each plot has a common top layer: (a) phantoms 2L3, 2L6, 2L9$, \mu_{a,1}=0.24[mm^{-1}]$ , (b) phantoms 2L2, 2L5, 2L8, $\mu_{a,1}=0.41[mm^{-1}]$, (c) phantoms 2L1, 2L4, 2L7, $\mu_{a,1}=0.6[mm^{-1}]$. The red-scheme graphs have a 1L3 bottom layer, The green-scheme graphs have a 1L2 bottom layer, and the blue-scheme graphs have a 1L1 bottom layer.


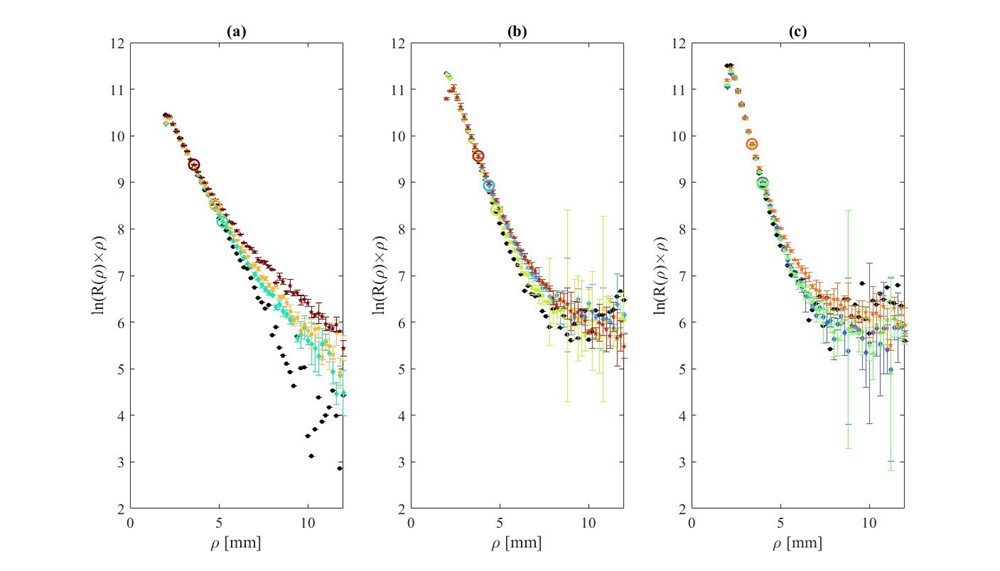


Figure 2. Bilayer phantoms results at 650 nm. (a) phantoms 2L3, 2L6, 2L9$, \mu_{a,1}=0.24[mm^{-1}]$ , (b) phantoms 2L2, 2L5, 2L8, $\mu_{a,1}=0.41[mm^{-1}]$, (c) phantoms 2L1, 2L4, 2L7, $\mu_{a,1}=0.6[mm^{-1}]$. The red-scheme graphs have a 1L3 bottom layer, The green-scheme graphs have a 1L2 bottom layer, and the blue-scheme graphs have a 1L1 bottom layer.


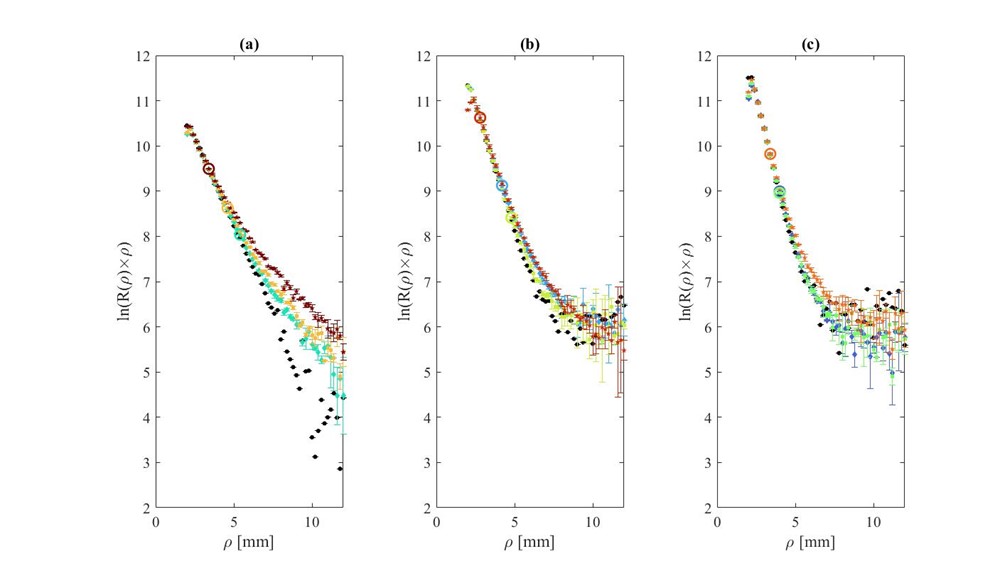


Figure 3. Bilayer phantoms results at 700 nm. (a) phantoms 2L3, 2L6, 2L9$, \mu_{a,1}=0.24[mm^{-1}]$, (b) phantoms 2L2, 2L5, 2L8, $\mu_{a,1}=0.41[mm^{-1}]$, (c) phantoms 2L1, 2L4, 2L7, $\mu_{a,1}=0.6[mm^{-1}]$. The red-scheme graphs have a 1L3 bottom layer, The green-scheme graphs have a 1L2 bottom layer, and the blue-scheme graphs have a 1L1 bottom layer.


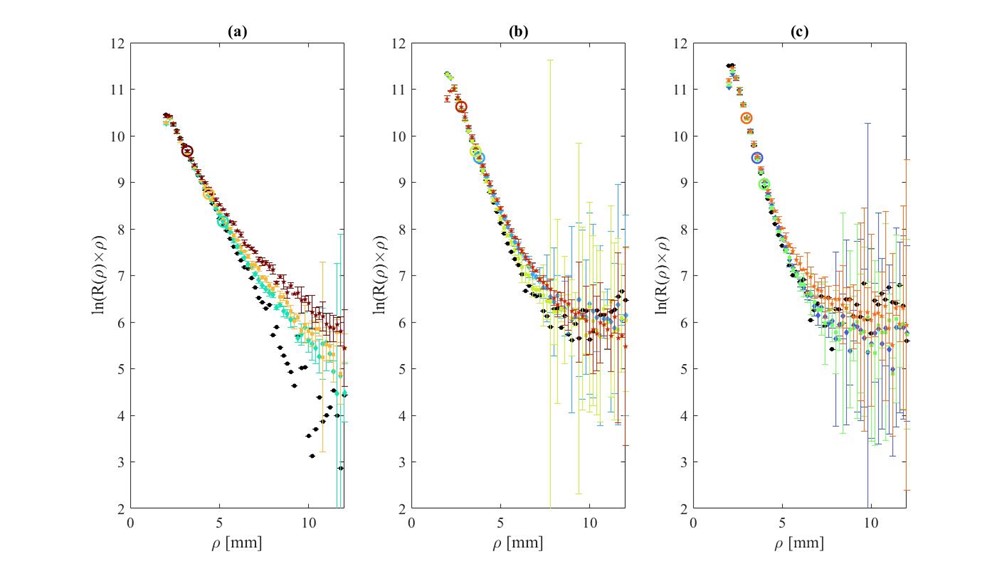


Figure 4. Bilayer phantoms results at 750 nm. (a) phantoms 2L3, 2L6, 2L9$, \mu_{a,1}=0.24[mm^{-1}]$, (b) phantoms 2L2, 2L5, 2L8, $\mu_{a,1}=0.41[mm^{-1}]$, (c) phantoms 2L1, 2L4, 2L7, $\mu_{a,1}=0.6[mm^{-1}]$. The red-scheme graphs have a 1L3 bottom layer, The green-scheme graphs have a 1L2 bottom layer, and the blue-scheme graphs have a 1L1 bottom layer.

1. **EXTRACTED** $\boldsymbol{C}_{\boldsymbol{p}}$ **AT 650nm, 700nm, AND 750nm**

From the experimental results, the $C_{p}$ was extracted for each wavelength, as shown in Figure 5. For all of the wavelengths, the observed behavior of the $C_{p}$ supports the theoretical prediction[1, 2].


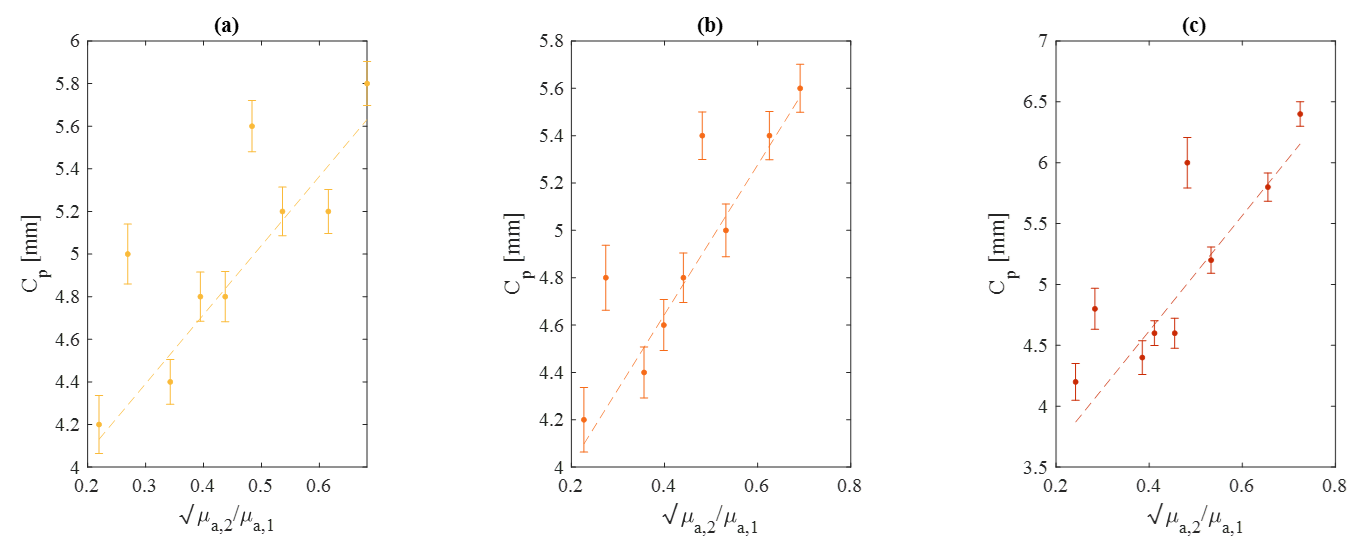


Figure 5. The $C_{p}$ extracted from the experimental data for (a) 650nm, (b) 700nm, (c) 750nm.

[1] H. Taitelbaum, S. Havlin, and G. H. Weiss, “Approximate theory of photon migration in a two-layer medium,” Applied optics*,* 28(12), 2245-2249 (1989).

[2] I. Dayan, S. Havlin, and G. H. Weiss, “Photon migration in a two-layer turbid medium A diffusion analysis,” Journal of Modern Optics*,* 39(7), 1567-1582 (1992).
